# Bronchoscopic Removal of Aspergilloma in a 26‐Year‐Old Man

**DOI:** 10.1002/rcr2.70696

**Published:** 2026-08-02

**Authors:** Chun‐Hao Wang, Lih‐Yu Chang, Jann‐Yuan Wang

**Affiliations:** ^1^ Department of Internal Medicine National Taiwan University Hospital Taipei Taiwan; ^2^ Department of Internal Medicine National Taiwan University Hospital Hsin‐Chu Branch Hsin‐Chu Taiwan

**Keywords:** aspergilloma, bronchoscopy, chronic pulmonary aspergillosis, fungal ball, pulmonary tuberculosis

## Abstract

A 26‐year‐old man with recurrent pulmonary tuberculosis and prior left pneumonectomy presented with progressive dyspnea. Chest computed tomography revealed a cavitary fungal ball in the right upper lobe, and elevated Aspergillus fumigatus IgG confirmed chronic pulmonary aspergillosis. Despite oral voriconazole, the cavity enlarged and surgery was contraindicated due to severely limited lung function. Bronchoscopic removal was performed using an ultrathin bronchoscope, two flexible bronchoscopes of increasing diameter, biopsy forceps, a Fogarty catheter and suction. The friable fungal mass was fragmented and completely extracted over two sessions. Histopathology and culture confirmed Aspergillus fumigatus. At 18‐month follow‐up, imaging showed complete resolution of the fungal ball with cavity‐wall thinning and no recurrence, accompanied by symptomatic improvement and a 45% decline in Aspergillus fumigatus IgG. Bronchoscopic removal may represent a feasible minimally invasive option in carefully selected, medically refractory, surgically ineligible patients, though durability remains unproven from a single case.

## Graphical abstract

A 26‐year‐old man with posttuberculous aspergilloma and a prior contralateral pneumonectomy remained refractory to voriconazole but was ineligible for surgery. The friable fungal ball was fragmented and completely extracted bronchoscopically over two sessions using progressively larger bronchoscopes, forceps, a Fogarty catheter and suction. No recurrence occurred at 18 months.

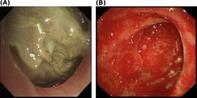

## 1 Introduction

Chronic pulmonary aspergillosis (CPA) is a progressive fungal infection caused by Aspergillus species, most commonly developing in the setting of preexisting structural lung disease such as posttuberculous cavities, bronchiectasis or prior pneumonectomy [1]. Aspergilloma, a classic manifestation of CPA, consists of a fungal ball—a conglomeration of hyphae, mucus and cellular debris—residing within a preformed cavity [2]. Standard treatment includes long‐term antifungal therapy with voriconazole or itraconazole; however, medical therapy alone frequently fails to achieve cure, and surgical resection remains the definitive treatment in eligible patients [3]. In individuals with severely compromised lung function, surgery carries prohibitive risk, leaving few therapeutic alternatives. Herein, we report a case of medically refractory aspergilloma successfully managed by bronchoscopic removal as a minimally invasive alternative.

## 2 Case Report

A 26‐year‐old man with a history of recurrent pulmonary tuberculosis and prior left pneumonectomy for refractory hemoptysis presented with progressive dyspnea. Chest computed tomography (CT) revealed a large cavitary lesion containing an intra‐cavitary soft‐tissue mass with characteristic ‘ball in hole’ and air‐crescent signs in the right upper lobe (Figure 1A). Serological testing demonstrated markedly elevated Aspergillus fumigatus IgG levels (≥ 200 AU/mL), consistent with a diagnosis of CPA. Despite treatment with oral voriconazole, the cavity progressively enlarged over several months. Given the patient's severely limited residual lung function following contralateral pneumonectomy, surgical resection was deemed infeasible.

**FIGURE 1:**
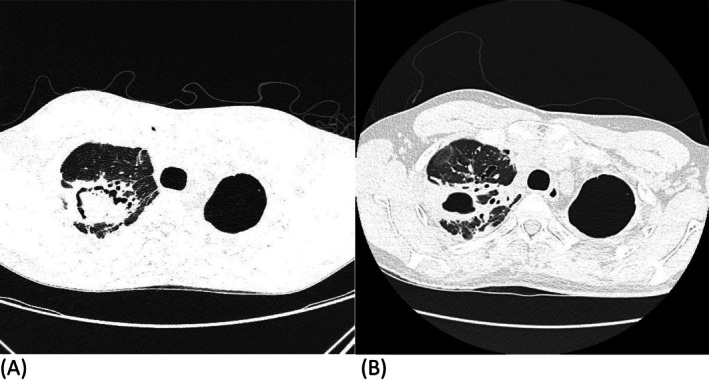
Chest computed tomography performed before (A) and after (B) bronchoscopy. The prebronchoscopic image (A) demonstrates a large cavitary lesion containing an intra‐cavitary soft‐tissue mass with the characteristic ‘ball in hole’ and air‐crescent signs in the right upper lobe. The postprocedure image (B) shows marked cavity‐wall thinning at 18‐month follow‐up with no recurrence.

Bronchoscopic removal of the aspergilloma was performed under general anaesthesia to access the right upper lobe superior segment (RB1), using three bronchoscopes of increasing diameter: an ultrathin bronchoscope (Olympus BF‐XP260F, outer diameter 2.8 mm), followed by the Olympus BF‐P290 (outer diameter 4.2 mm) and Olympus BF‐Q290 (outer diameter 4.8 mm). High‐flow nasal cannula oxygen therapy was administered throughout the procedure to maintain adequate oxygenation, and a Fogarty catheter was prepared in anticipation of potential major airway bleeding. Bronchoscopic views before and after the procedure are shown in Figure 2. The fungal ball was found to be relatively soft and friable, precluding en‐bloc removal with forceps alone. The mass was initially fragmented using endobronchial biopsy forceps, and a Fogarty catheter was subsequently used to provide traction and mobilize the fungal ball, followed by repeated bronchoscopic suction to remove fragmented material until complete clearance was achieved (Figure 2B). The procedure was completed over two separate sessions, with a cumulative procedural duration of approximately 4 h. No significant haemorrhage or pneumothorax occurred during either session.

**FIGURE 2:**
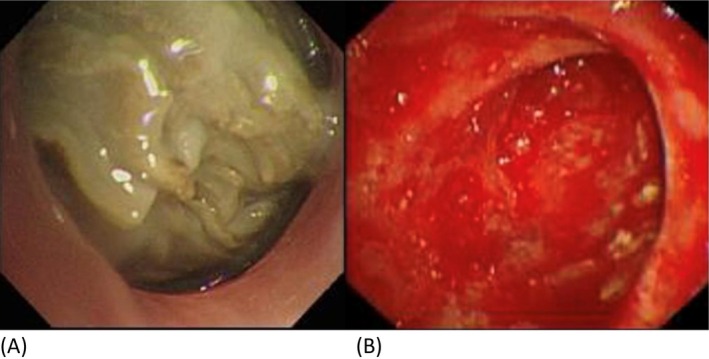
Bronchoscopic views of the right upper lobe superior segment (RB1) before (A) and after (B) removal of the fungal ball. The fungal ball was relatively soft and friable, requiring a combined forceps‐and‐suction technique for complete extraction.

Histopathological examination of the extracted material demonstrated fragments of fungal ball (haematoxylin and eosin stain, 40×), with uniform, septated hyphae exhibiting regular acute‐angle dichotomous branching at higher magnification (400×), findings consistent with Aspergillus species (Figure 3). Fungal culture confirmed Aspergillus fumigatus. The patient tolerated the procedure without immediate complications. Follow‐up chest CT at 18 months demonstrated complete disappearance of the intracavitary fungal ball with progressive cavity‐wall thinning and no evidence of recurrence (Figure 1B), with continued antifungal maintenance therapy. Clinically, the patient's respiratory symptoms improved after the procedure. In addition, Aspergillus‐specific IgG levels decreased substantially during follow‐up, with Aspergillus fumigatus IgG decreasing from 177 to 97.5 AU/mL and Aspergillus niger IgG decreasing from 33.6 to 21.6 AU/mL.

**FIGURE 3:**
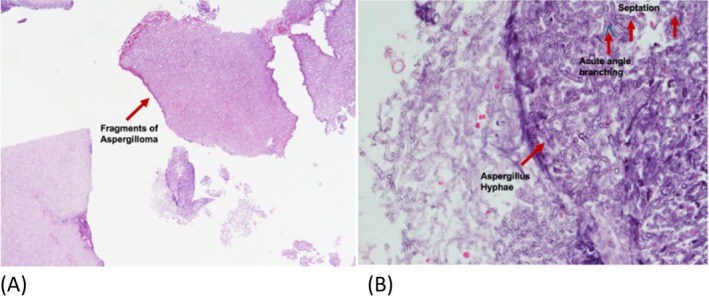
Histological findings of the bronchoscopic biopsy specimen from the intra‐cavitary mass. (A) Fragments of fungal ball (haematoxylin and eosin stain, 40×). (B) Uniform, septated hyphae with regular acute‐angle dichotomous branching consistent with Aspergillus fumigatus (haematoxylin and eosin stain, 400×).

## 3 Discussion

This case demonstrates that bronchoscopic removal can be a feasible minimally invasive therapeutic option for carefully selected patients with medically refractory CPA who are not candidates for surgical resection. Aspergilloma typically develops within preexisting pulmonary cavities, frequently in patients with prior tuberculosis, and these individuals often have severely compromised lung function that precludes conventional surgery [2, 3]. In our patient, prior contralateral pneumonectomy rendered surgical intervention prohibitively dangerous.

The bronchoscopic approach has been reported in limited case series, employing various instruments including forceps, cryoprobes, and laser ablation [4, 5]. A key technical challenge encountered in this case was the soft, friable consistency of the fungal ball, which necessitated a combined forceps‐and‐suction technique rather than simple grasping. A Fogarty catheter additionally provided traction during debulking and was kept available as a precautionary measure for potential bronchial tamponade in case of major bleeding. The use of bronchoscopes of progressively larger calibre enabled access to the segmental bronchus leading to the cavity while accommodating the instruments required for fragmentation and suction. Importantly, successful complete extraction was achieved without significant haemorrhage or pneumothorax.

The sustained 18‐month radiological response, characterized by progressive cavity‐wall thinning and absence of recurrence, is encouraging, though longer‐term studies in larger cohorts are needed to confirm durability. Bronchoscopic removal of aspergilloma is a technically demanding procedure that carries risks including massive hemoptysis, pneumothorax, transient hypoxemia, airway obstruction caused by fragmented fungal material and incomplete removal. It should therefore be performed only by experienced bronchoscopists, with appropriate preparation and immediate availability of measures to control major airway bleeding, and with careful patient selection. In conclusion, bronchoscopic removal may represent a feasible minimally invasive option for carefully selected patients with medically refractory aspergilloma who are not surgical candidates, though its efficacy and durability remain unproven based on a single case.

## Funding

The authors have nothing to report.

## Consent

The authors declare that written informed consent was obtained for the publication of this manuscript and accompanying images using the form provided by the Journal.

## Conflicts of Interest

The authors declare no conflicts of interest.

## Data Availability

The data that support the findings of this study are available on request from the corresponding author. The data are not publicly available due to privacy or ethical restrictions.
